# DNA double-strand break genetic variants in patients with premature ovarian insufficiency

**DOI:** 10.1186/s13048-023-01221-2

**Published:** 2023-07-10

**Authors:** Xuechun Ding, Xiaowei Gong, Yingying Fan, Jinghe Cao, Jingyu Zhao, Yixin Zhang, Xiaomei Wang, Kai Meng

**Affiliations:** 1 Collaborative Innovation Center for Birth Defect Research and Transformation of Shandong Province, Jining Medical University, Jining, China; 2 College of Second Clinical Medical, Jining Medical University, Jining, China; 3 Affiliated Hospital of Jining Medical University, Jining, China; 4 College of Basic Medicine, Jining Medical University, Jining, China; 5 Lin He’s Academician Workstation of New Medicine and Clinical Translation, Jining Medical University, Jining, China

**Keywords:** Premature ovarian insufficiency, DNA double-strand breaks, Homologous recombination, Non-homologous end joining, Infertility

## Abstract

Premature ovarian insufficiency (POI) is a clinically heterogeneous disease that may seriously affect the physical and mental health of women of reproductive age. POI primarily manifests as ovarian function decline and endocrine disorders in women prior to age 40 and is an established cause of female infertility. It is crucial to elucidate the causative factors of POI, not only to expand the understanding of ovarian physiology, but also to provide genetic counselling and fertility guidance to affected patients. Factors leading to POI are multifaceted with genetic factors accounting for 7% to 30%. In recent years, an increasing number of DNA damage-repair-related genes have been linked with the occurrence of POI. Among them, DNA double-strand breaks (DSBs), one of the most damaging to DNA, and its main repair methods including homologous recombination (HR) and non-homologous end joining (NHEJ) are of particular interest. Numerous genes are known to be involved in the regulation of programmed DSB formation and damage repair. The abnormal expression of several genes have been shown to trigger defects in the overall repair pathway and induce POI and other diseases. This review summarises the DSB-related genes that may contribute to the development of POI and their potential regulatory mechanisms, which will help to further establish role of DSB in the pathogenesis of POI and provide theoretical guidance for the study of the pathogenesis and clinical treatment of this disease.

## Introduction

Premature ovarian insufficiency (POI), also known as premature ovarian failure, is characterised by the decline of ovarian function and associated endocrine disorders in women prior to age 40, characterised by elevated serum follicle-stimulating hormone levels with associated decreased estradiol levels and accompanied by menstrual disorders, including primary and secondary amenorrhea [1]. In some clinical cases, POI patients not only suffer from menstrual disorders, infertility and other symptoms of diminished reproductive function, but may also have osteoporosis and other unrelated complications such as autoimmune disease and cardiovascular disease [2, 3], which may seriously affect both physical and mental health. The incidence of POI is age-related, with reported rates of approximately 1:10,000 under age 20, 1:1,000 under age 30 and 1:100 under age 40 [4]. Three mechanisms are known to contribute to POI: a limited number of oocytes, expedited follicular atresia and declined folliculogenesis [1]. POI is not rare in clinical practice and its incidence is increasing every year. Although the specific pathogenic mechanisms of POI are not clear, it is known that genetics, autoimmunity, iatrogenic injury, enzyme deficiencies and environmental factors such as psychological stress and environmental toxins may contribute to the development of POI [2]. The majority of patients, however, have no known aetiology and are referred to as idiopathic POIs. Patients with idiopathic POI are subdivided into familial and sporadic cases based on the presence or absence of a family history of the disease [3, 5].

Notably, genetic factors account for 7% to 30% of all causative factors of POI and have been a hotspot for POI research [6–9]. Genetic factors are divided into two categories: chromosomal aberrations and gene mutations. Chromosomal aberrations are divided into X-chromosome aberrations and autosomal aberrations. The common chromosomal aberrations are numerical (such as X monosomy, 45, X/46, XX mosaicisms, X trisomy) and morphological (such as chromosomal deletions, translocations and rearrangements). Chromosomal aberrations often lead to syndromic POI, in which the patient has the typical symptoms of POI, such as ovarian dysfunction and hypogonadotropic hypoestrogenism, in addition to other heritable symptoms caused by the chromosomal aberration. Syndromic POIs with a high incidence include Turner syndrome, autoimmune polyendocrinopathy syndrome type I, galactosemia and carbohydrate-deficient glycoprotein syndromes and ataxia telangiectasia [10], while genetic mutations often lead to isolated POI. Previous studies have classified more POI-causing genes, including folliculogenesis genes, meiosis and DNA repair genes and granulosa cell proliferation and differentiation genes [4, 11]. Among them, DNA double-strand breaks (DSB), which are mainly repaired by homologous recombination (HR) and non-homologous end joining (NHEJ), are the most devastating type of DNA damage [12, 13]. Mutations in key genes during this process can affect the normal physiological function of the ovary and can easily lead to follicular atresia or oocyte apoptosis [14], leading to POI. This review highlights the DSB-associated genes, some meiosis-specific genes, and specific mutations in these genes identified in recent years in patients with familial and sporadic POI providing future investigators with a better understanding of the potential pathogenic mechanisms of these genes in POI. This area of research will potentially lead to new ideas for the prevention of POI, early detection of POI and treatment of POI at the genetic level, further contributing to the knowledge and understanding of female reproductive health.

## DSBs

### The formation of DSB

DSB refers to the fact that both DNA strands of deoxyribonucleotide are broken, which is the most significant DNA damage. There are two types of DSBs formed by the human body, one is programmed DSB, which is involved in the first meiotic division, V(D)J rearrangement of lymphocytes and heavy chain class switch, neuronal gene expression and is necessary to promote genetic diversity and normal physiological functions. The other type is accidental DSB [15, 16].

The correct production of programmed DSBs in meiosis, is a prerequisite for homologous chromosomal gene recombination and is essential for maintaining ovarian function. The process of the formation of programmed DSBs is highly conserved and complex across species. The location of the introduced programmed DSB is mainly determined by the PRDM9 protein with methyltransferase activity, rather than appearing to be random [17]. The terminal C2H2 zinc finger domain of PRDM9 binds DNA and the SET domain trimethylates histone H3 at lysine 4 and lysine 36 (H3K4me3, H3K36me3) to determine the location of the recombination hotspot, namely, the DSB sites [13, 18, 19]. Subsequently, PRDM9 recruits topoisomerase VI (TopoVI) to the marked hotspot site. TopoVI consists of SPO11 and TopoVIBL, which together exert a topoisomeric-like reaction, severing the DNA double-strand through transesterification reaction and binding to the 5' end of the broken DNA strands [18, 20, 21]. In addition, a pre-DSB recombinosomes consisting of IHO1, MEI1, MEI4, REC114 and ANKRD31 assists the role of SPO11-TopoVIBL, the deletion or mutation of which leads to abnormal DSB formation [18, 22].

Accidental DSBs have two main sources, internal and external. The main endogenous factors that cause DSB are replication stress, reactive oxygen species (ROS) and special DNA structures such as telomeres and retrotransposons. Exogenous factors include ionising radiation, UV radiation, chemical drugs, etc. [16]. Ionising radiation damages the DNA double-strand directly or indirectly through energy deposition and production of ROS. In contrast, UV radiation and most chemical drugs generate and repair DNA adducts to convert them into DSBs. Some drugs such as camptothecin and trabectedin trigger DSBs by blocking transcription [23].

### Repair pathways for DSBs

Each cell of all organisms experience approximately 10 DSBs per day [24] and if not repaired or repaired incorrectly, the DSBs may increase the risk of certain diseases. Non-homologous end joining (NHEJ) and homologous recombination (HR) are two distinct DSB repair methods. NHEJ is the predominant DSB repair method in eukaryotic cells, directly ligating broken DNA ends through a series of polymerases, nucleases and ligases and is relatively error-prone [25]. There are two types of NHEJ: classical NHEJ (cNHEJ) and alternative NHEJ (Alt-NHEJ). Most researchers believe that Alt-NHEJ is a repair pathway that is initiated when cells lack key enzymes for cNHEJ such as Ku and Artemis and is specialized as it requires the presence of a homologous sequence of 2–20 nucleotides at the end of the DNA break to activate the repair process [26]. NHEJ can occur at any stage of the cell cycle, but is most active in G1 phase, whereas HR, which is more accurate for repair results, can only occur in S and G2 phases since the HR pathway requires sister chromatids as homologous templates [27, 28].

#### Homologous recombination

Certain excision of the broken DNA ends, is the first step of HR. This process is further divided into two stages: the first stage is the excision of a portion of the oligonucleotide at the 5' end of the DNA break using CtIP and MRE11-RAD50-NBS1 (MRN) complex [29], during meiosis I, the excised portion of the oligonucleotide binds to SPO11, the DSB formation protein.; the second stage utilises EXO1 which has 5'–3' exonuclease activity and WRN1-DNA2 complex with helicase and endonuclease activity to further excise the nucleotide at the 5' end, resulting in long 3′ssDNA overhangs [13, 30, 31]. Immediately after the second stage, RPA (a heterotrimer consisting of RPA1, RPA2 and RPA3), which has a high affinity for ssDNA, binds to the 3′ssDNA overhangs, thus preventing it from forming secondary structures or from being degraded by nucleases [32, 33]. Subsequently, with the assistance of BRCA2, RAD51 replaces RPA and binds ssDNA to form the RAD51-ssDNA nucleoprotein filament [34]. In meiosis, RAD51 plays only an auxiliary role and it is mainly the RAD51 paralog DMC1 that binds to ssDNA [13, 35]. RAD51-ssDNA nucleoprotein filament invades dsDNA and searches for homologous sequences to form D-loop structures [30]. Factors that promote nucleoprotein filament formation and single-strand invasion include RAD51 paralogs (RAD51B/C/D and XRCC2/3) and the heterodimeric protein HOP2-MND1 [13]. On the basis of the structure of proteins and intermediates dealing with the D-loop structure, HR is divided into 2 main sub-pathways: the synthesis-dependent strand annealing and the double Holliday junction (dHJ) pathway [36]. Many complexes are involved in the processing and stabilisation of HR intermediates, such as MCM8-MCM9 helicase complex, MSH4-MSH5 heterodimer, HFM1, RECQL4, BLM, etc. [13]. The last process is the synthetic repair of DNA and intermediate resolution, which requires the removal of RAD51 (and DMC1) from the D-loop, followed by the synthesis of the complementary strand from the 3' end of the invasion strand using DNA polymerase [37]. The integral HR process is dynamic and is accomplished by the action of interlinked enzymes and regulatory proteins that delicately regulate ssDNA and nucleoprotein filament formation, stabilisation, resolution and homologous strand search [34].

#### Non-homologous end joining

The NHEJ process is relatively simple compared to HR, but it is the predominant DSB repair method in eukaryotic cells. In the classical NHEJ pathway, the ring-shaped Ku70/80 heterodimer first monitors the formation of DSBs and then rapidly binds to broken DNA ends to form Ku: DNA complexes [27]. The Ku: DNA complex resembles a platform for assembling NHEJ-related enzymes and proteins that recruit and activate DNA-PKcs, XRCC4, XLF, PAXX, LIG4 and other constituents [12, 38, 39]. DNA-PKcs phosphorylate a variety of NHEJ key factors, including itself and its kinase activity is essential for efficient end joining [25, 39]. The NHEJ process also has a key end processing enzyme, Artemis, with 5'–3' exonuclease activity and endonuclease activity [40]. Artemis forms a DNA-PKc-Artemis complex with DNA-PKcs that not only efficiently cleaves the structure of the complex broken DNA ends, but also ligates in conjunction with the important DNA ligase4 (LIG4), thereby facilitating the interaction between LIG4 and DNA-PKcs [27]. In addition to Artemis, the intricate processing of DNA end resection, processing and synthesis is inseparable from the action of tyrosyl-DNA phosphodiesterase 1 (TDP1), polynucleotide kinase (PNK) and DNA polymerases [41, 42]. When Artemis and other enzymes finish processing the broken DNA ends, the core ligase complex XRCC4-XLF-LIG4 begins to mediate the joining of the two broken ends [43]. Among them, XRCC4 stabilises LIG4 and enhances its linkage activity by increasing the adenylation efficiency of LIG4 [44].

The specific HR and NHEJ processes are shown in Figure A and B. Once the enzymes or proteins related to the DSB repair pathway are missing or abnormal in the body, it may cause errors in repair, leading to the accumulation of DSBs and eventually to disease.

## Cohesin and synaptonemal complex

Human cohesin complex is ring-like, involving chromosome separation and DSB repair in mitosis and meiosis [45]. During repair of meiotic programmed DSBs, intact cohesin complex prevents sister chromatids from being incorrectly separated during the first meiotic division. Cohesin proteins are essential for the assembly of the axial elements of the synaptonemal complex (SC) and facilitate the correct formation of the synaptonemal complex [46]. The meiosis-specific cohesin complex contains a total of four subunits: STAG3, REC8, RAD21L and SMC1B [46–48]. Relatively, *STAG3* mutations are more frequent in POI patients, while the other three genes cause fewer cases of female reproductive disorders. *RAD21L* is more frequently associated with male reproductive disorders.

Synaptonemal complex (SC) is a meiosis-specific multiprotein complex located between homologous chromosomes, that participates in programmed DSB-dependent homologous chromosome synapsis and fragment exchange and promotes genetic diversity [49]. The complete SC consists of two lateral elements (LE) arranged in parallel, a central element (CE) and transverse filaments (TF) [50]. The transverse filaments connecting the LE are mainly composed of SYCP1 protein, the LE are composed of SYCP2 and SYCP3, and the CEs are composed of SYCE1, SYCE2, SYCE3, TEX12 and C14orf39 [51]. The correct formation and regulation of SC is an important part of HR in meiosis, while an error in its formation or regulation can lead to infertility or miscarriage.

## POI causing genes

Genes and candidate genes that can cause POI have been identified which regulate multiple processes in female reproduction including the growth and development of follicles at all levels, mitosis and meiosis, DNA damage repair, etc. Below we summarise the genes associated with the formation and repair of DSBs that can lead to POI and the detailed mutations identified in patients with POI. (Table 1).

**Table 1:** The formation and repair of DSBs mutations in POI patients

Gene	Location	Function	Mutation	Genotype	Type of Variants	Amenorrhea	Familial/Sporadic	References
*PRDM9*	5p14.2	determines the positioning of the recombinant hotspots	c.229C > T, p. Arg77*	het		secondary	sporadic	[14]
			c.638 T > G, p.Ile213Ser	het		secondary	sporadic	
			c.677A > T, p.Lys226Met	het		secondary	sporadic	
*ANKRD31*	5q13.3	regulates the assembly of pre-DSB recombinsomes	c.1565-2A > G	het	splice site	secondary	sporadic	[14]
			c.985C > T, p.Gln329*	het	nonsense	secondary	sporadic	
*NBN*	8q21.3	participates in DNA end resection	c.871C > T, p.(Gln291*)	hom	nonsense	primary	sporadic	[52]
			c.657_661del5	hom	Slavic founder	secondary	sporadic	[53]
*HSF2BP*	21q22.3	promotes homologous chromosomes synapsis and the formation of crossover	S167L	hom	missense	secondary	familial	[54]
			c.382 T > C, p.C128R	hom	missense	secondary	sporadic	[55]
			c.557 T > C, p.L186P	hom	missense	secondary	sporadic	
*EXO1*	1q43	participates in DNA end resection	c.155C > G, p.Thr52Ser	het	missense	primary	sporadic	[56]
			c.668G > A, p.Gly223Asp	het	missense	primary	sporadic	
*PSMC3IP*	17q21.2	promotes the formation of nucleoprotein filament indirectly and strand exchange	c.496_497delCT, p.R166Afs	com het	Deletion	primary	sporadic	[57]
			c.430_431insGA, p.L144*		Insertion			
			c.489 C > G, p.Tyr163Ter	hom	stop gain		familial	[58]
			c.597 + 1G > T	com het	splicing		familial	[59]
			c.268G > C, p.D90H					
			c.206_208delAGA, p.Lys69del	com het	deletion		familial	[60]
			c.189 G > T, p.Lys63Asn		missense		familial	
*BRCA2*	13q13.1	mediates the binding of recombinase RAD51 to ssDNA	c.7579delG, p.V2527X	com het	deletion	primary	familial	[61]
			c.9693delA, p. S3231fs16*		deletion			
			c.8524C > T, p.R2842C	hom	missense	primary	familial	[62]
*DMC1*	22q13.1	binds ssDNA during meiosis	c.106G > A, p.Asp36Asn	hom	missense	secondary	familial	[63]
*MEIOB*	16p13.3	MEIOB-SPATA22 complexes can be recruited to DSB sites, promoting intact synapsis and the foramtion of crossover	c.1218G > A	hom	splicing	secondary	familial	[64]
			c.683-1G > A	hom	splicing	secondary	familial	[65]
*SPATA22*	17p13.2		c.400C > T, p.R134X	hom	nonsense	normal	familial	[66]
			c.900 + 1G > A	com het	splicing	secondary	sporadic	
			c.31C > T, p.R11X					
*SPIDR*	8q11.21	promotes the formation of nucleoprotein filament	c.839G > A, p.W280*	hom	stop-gain	primary	familial	[67]
			c.814C > T, R272*	hom	nonsense	primary	sporadic	[68]
*MSH4*	1p31.3	MSH4-MSH5 heterodimer can bind and stabilize the Holliday intermediates, promoting the formation of crossover	c.2355 + 1G > A	hom	donor splice-site	secondary	familial	[69]
			c.2261C > T, p.Ser754Leu	hom	missense	secondary	familial	[70]
			c.2198C > A, p.Ser733Ter	hom	stop-gain	secondary	sporadic	[71]
*MSH5*	6p21.33		c.1459G > T, p.D487Y	hom	missense	secondary	familial	[72]
			c.1057C > A, p.L353M	het		secondary	sporadic	
			c.1459G > T, p.D487Y	het		secondary	sporadic	
			c.2107 A > G, p.I703V	het		secondary	sporadic	
			c.C1051G, p.R351G	het	missense	primary	sporadic	[73]
			c.2063 T > C, p.I688T	com het		secondary	sporadic	[74]
			c.604G > C, p.G202R					
*MCM8*	20p12.3	MCM8-MCM9 hellicase complex participates in DNA end resection and promotes intermediates processing	c.446C > G, p.P149R	hom	missense	primary	familial	[75]
			c.1954-1G > A	hom	splice	primary	familial	[76]
			c.1469-1470insTA	hom	frameshift	primary	familial	
			c.464G > A, p.Cys155Tyr	het		primary	sporadic	[77]
			c.548A > G, p.Asn183Ser	het		primary	sporadic	
			c.1334G > A, p.Arg445Gln	het	missense	primary	sporadic	
			c. A950T, p. H317L	het	missense	primary	sporadic	[78]
			c. A1802G, p. H601R	het	missense	secondary	sporadic	
			c. 482A > C, p.His161Pro	hom	missense	primary	familial	[79]
			c.89A > C, p.K30T	com het			familial	[80]
			c.1330A > G, p.I444V					
			c.925C > T, p.R309*	hom			familial	
			c.925C > T, p.R309*	hom	nonsense	primary	familial	[81]
			c.724 T > C, p.C242R	com het	missense	primary	familial	[82]
			c.1334C > A, p.S445*		termination			
			c.351_354 delAAAG, p. K118Efs*5	hom	frameshift	primary	familial	[83]
*MCM9*	6q22.31	MCM8-MCM9 hellicase complex participates in DNA end resection and promotes intermediates processing	c.1732 + 2 T > C,	hom	splicing	primary	familial	[84]
			c.394C > T, p.Arg132*	hom	insertion	primary	familial	
			c.397 T > C, p.Thr139Ala	het		secondary	sporadic	[77]
			c.2422G > A, p.Val808Ile	het	missense	secondary	sporadic	
			c.1784C > G, p.Thr595Arg	het	missense	primary	sporadic	
			c.1651C > T, p.Gln551*	hom	stop gain	primary	sporadic	
			c.1483G > T, p.E495*	hom	nonsense	primary	familial	[85]
			c.220C > T, p.R74*	hom			familial	[80]
			c.1473dup, p.Thr492TyrfsTer4	hom	frameshift		familial	[86]
			c.2059 T > C, p.F687L	com het	missense	primary	sporadic	[87]
			c.3223C > T, p.P1075S		missense			
			c.1163C > A, p.T388N	het		secondary	sporadic	
			c.C1423T, p.L475F	het	missense	secondary	sporadic	[88]
			c.T2921C, p.L974S	het	missense	secondary	sporadic	
			c.G3388A, p.A1130T	het	missense	secondary	sporadic	
			c.2488G > A, p.A830T	het	missense	secondary	sporadic	[89]
			c.1157C > T, p.T386M	com het		secondary	sporadic	[74]
			c.1291A > G, p.M431V				sporadic	
*HFM1*	1p22.2	promotes homologous chromosomes synapsis and the formation of crossover	c.1686–1 G → C	com het			familial	[90]
			c.2651 T → G, p.Ile884Ser					
			c.2206 G → A, p.Gly736Ser	com het			sporadic	
			c.3929_3930 delinsG, p.Pro1310Arg fs*41		frameshift			
			c.148G > A, p.Glu50Lys	het	missense		sporadic	[91]
			c.1241A > C, p.His414Pro	het	missense		sporadic	
			c.2325C > A, p.Phe775Leu	het	missense		sporadic	
			c.3367 T > C, p.Ser1123Pro	het	missense		sporadic	
			c.3580C > T,p.Arg1194Cys	het	missense		sporadic	
			c.1686-1G > C	het	splice-site		sporadic	
			c.3470G > A	het	missense	secondary	familial	[91]
			c.3100G > A, p.G1034S	com het	missense	secondary	sporadic	[92]
			c.1006 + 1G > T		splice-site			
*ERCC6*	10q11.23	promotes HR while suppresses NHEJ	c.C1769C > T, p. P590L	het	missense	secondary	sporadic	[74]
*(CSB-PGBD3)*			c.643G > T, p. E215X	het	nonsense	secondary	sporadic	[93]
			c.3166G > A, p. V1056I	het	missense	secondary	sporadic	
*STAG3*	7q22.1	maintains cohesin stability, ensures correct assemblage and segregation of homologous chromosomes	c.968delC, p.F187fs*7	hom	frameshift	primary	familial	[94]
			c.1947_48dupCT, p.(Y650Sfs*22)	hom	duplication	primary	familial	[95]
			c.1573 + 5G > A, p.Leu490Thrfs*10	hom	donor splice site	primary	familial	[96]
			c.291dupC, p.Asn98Glnfs*2	com het	loss-of-function	primary	sporadic	[87, 97]
			c.1950C > A, p.Tyr650*		loss-of-function		sporadic	
			c.3052delC, p.Arg1018Aspfs*14	com het	deletion	primary	familial	[98]
			c.659 T > G, p.Leu220Arg		missense		familial	
			c.877_885del, p.293_295del	hom	in-frame	primary	familial	[99]
			c.891_893dupTGA, p.297_298insAsp	hom	in-frame	primary	familial	
			c.962G A, p.Arg321His	hom	missense	primary	familial	[100]
			c.962G > A, p.(Arg321His)	hom	missense	primary	sporadic	[101]
			c.1336G > T, p.(Glu446Ter)	hom	nonsense	primary	familial	[102]
			c.659 T > G, p.Leu220Arg	het		primary	sporadic	[103]
			c.938A > T, p.Tyr313Phe	com het		primary	sporadic	
			c.1999C > T, p.Arg667Cys			primary	sporadic	
			c.2473C > G, p.Leu825Val	het		primary	sporadic	
			c.2612G > A, p.Arg871His	het		primary	sporadic	
			c.3381_3384delAGAA, p.Glu1128Metfs*42	hom	deletion	primary	sporadic	[104]
			c.1942G > A, p. Ala648Thr	hom	missense		familial	[105]
			c.1951_1953del, p. Leu652del	hom	in-frame deletion		familial	
			c.2773delT, p.Ser925Profs*6 del	hom	deletion	primary	sporadic	[106]
*REC8*	14q12	part of the cohesin complex	c.1035_1036dup p.Glu346Glyfs*72	Com het	frameshift	secondary	familial	[107]
			c.624 + 1 G > A p.?		splicing			
*SMC1B*	22q13.31	part of the cohesin complex	c.662 T > C, I221T	het	missense	secondary	sporadic	[108]
			c.3530A > T, Q1177L	het	missense	primary	sporadic	
*C14orf39*	14q23.1	part of the synaptonemal complex	c.204_205del, p.His68Glnfs*2	hom	frameshift	secondary	familial	[109]
			c.508C > T, p.Arg170*	hom	nonsense	secondary	sporadic	[110]
*SYCE1*	10q26.3	part of the synaptonemal complex	c.613C > T, p.Gln205*	hom	nonsense	primary	familial	[111]
				hom	deletion	secondary	familial	[112]
			c.475G > A, p.Glu159Lys	com het	missense	secondary	sporadic	[110]
			c.689_690del, p.Phe230Serfs*21		frameshift			
*NHEJ1*	2q35	stabilizes and enhances the ligation activity of the XRCC4-LIG4 complex	c.532C > T, p.R178*	het	nonsense	secondary	familial	[113]
			c.500A > G, p.Y167C	het	missense	secondary	sporadic	

*Hom* homozygous, *Het* heterozygous, *Com het* compound heterozygous，*translation termination codon

### POI caused by genes related to DSB formation

#### *PRDM9* (PR-Domain Containing Protein 9) and *ANKRD31* (Ankyrin Repeat Domain 31)

Among the proteins involved in programmed DSB formation, PRDM9 and ANKRD31 have been previously reported to contribute to the POI phenotype. In 2021, Wang et al. analysed exome sequencing data from 1030 patients with POI and identified three heterozygous *PRDM9* mutations and two *ANKRD31* mutations in seven patients who developed secondary amenorrhea prior to age 38 [14]. PRDM9 catalyses lysine trimethylation at positions 4 and 36 of histone H3 using the methyltransferase activity of its PR/SET structural domain, thereby identifying the location of recombination hotspots [17, 114, 115]. All three *PRDM9* mutations in this report, impaired their methyltransferase activity, leading to abnormal histone trimethylation and affecting the localisation of recombination hotspots and programmed DSB formation. In the *Prdm9-/-* mouse model, there is an increased probability of unrepaired DSBs and a failure of synapsis and meiotic arrest in pachytene, with both male and female mice exhibiting infertility [116]. In particular, Hayashi et al. found almost no germ cells in the ovaries of *Prdm9* -/- neonatal female mice and little to no follicles at any stage in the five-week-old ovaries, suggesting that deletion or mutation of this gene would have a serious impact on mouse reproduction [117]. A study by Christopher L. Baker et al. found that in *Prdm9* ± mice, the number of recombination hotspots determined by *Prdm9* was reduced and the chance of meiotic errors was increased, leading to decreased reproductive function. After knockdown of *Prdm9*, while DSBs can still form, most of these DSBs are located at Prdm9-independent H3K4me3 sites, such as promoters and enhancers, which may lead to meiotic arrest [118].

In the study of Wang et al., two different types of *ANKRD31* mutations were also found. Both mutations weakened the interaction between ANKRD31 and REC114 and were unable to further stabilise and regulate the binding of downstream DSB-forming proteins to chromatin. Mice with knocked out *Ankrd31* have been reported to result in an increase in the number of DSBs and the enabling of the default DSB site, which also results in decremental efficiency of the regulation of DSB formation and may be responsible for the loss of synapsis and the delay in DSB repair [119]. The study by Michiel Boekhout et al., reported that *Ankrd31-/-* mice, although fertile, had greatly reduced numbers of primordial follicles and oocytes and smaller ovarian volumes than wild-type mice. That is, they exhibited POI-like characteristics [120]. Many of the genes known to be involved in the formation and regulation of DSB are known to contribute to male infertility [121], yet the research in female reproduction regarding the expression of these genes in POI patients is currently lacking and warrants further investigation.

### POI caused by genes related to HR

**Fig. 1:**
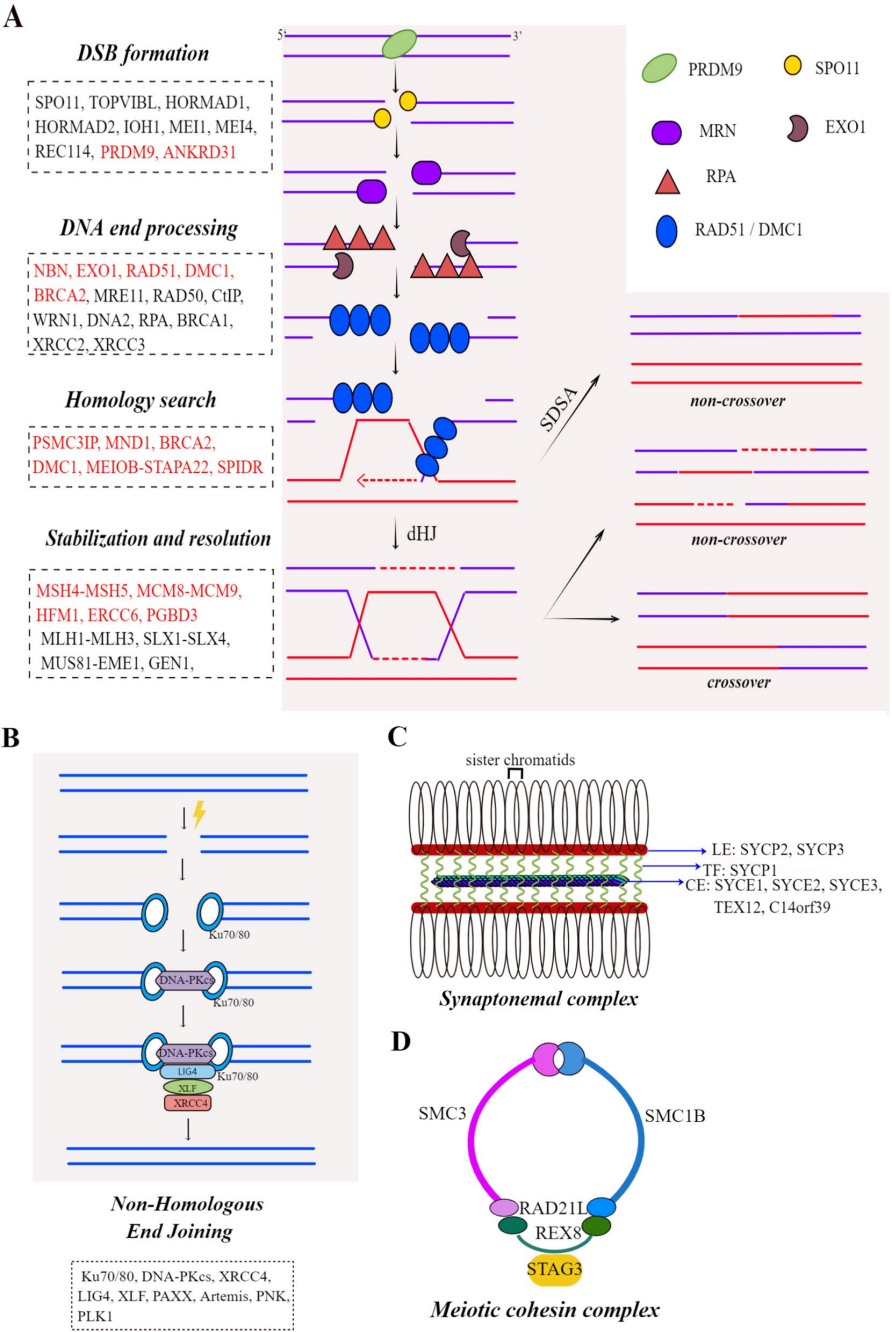
(By Figdraw(www.figdraw.com)): **A** The main processes of programmed DSB formation and homologous recombination (HR) repair. The genes involved in each step are shown in the boxes and genes associated with POI pathogenesis are marked in red. **B** The main processes and genes involved in non-homologous end joining repair (NHEJ). **C** Schematic diagram of the structure and protein composition of the synaptonemal complex. **D** Schematic diagram of the structure and protein composition of the cohesin complex in meiosis

The basic process of HR is divided into the following four stages: DNA end processing, homology search, intermediate formation and stabilisation and intermediate resolution [32, 51]. DNA end processing includes two processes, DNA end resection and formation of RAD51-ssDNA nucleoprotein filaments. The cohesin complex and the synaptonemal complex (SC), essential multiprotein structures formed during the HR process and the genes associated with them, were also found to be involved in the pathogenic process of POI. The specific HR processes, structures of cohesin complex and SC and genes associated with the pathogenesis of POI are shown in Fig. 1.

#### POI caused by genes related to DNA end processing

##### *NBN* (Nibrin)

NBN, also known as NBS1, binds to MRE11 and RAD50 to form the MRN complex, which is involved in the first step of HR, termed as DNA end resection. NBN itself has no enzymatic activity or ability to bind DNA, but rather acts as a recruiter and coordinator. NBN phosphorylation is important for the rapid formation of the MRN complex at the site of the DNA lesions. Mutations in *NBN* can lead to chromosomal instability and trigger immunodeficiency or susceptibility to cancer [52, 122–126]. In 2018, Elena J. Tucker et al. identified an NBN homozygous nonsense mutation in the gene of a POI patient without a familial genetic background [52]. Anna Szeliga et al. previously reported on a 23-year-old Polish woman with Nijmegen breakage syndrome who presented with secondary amenorrhea, elevated serum gonadotropin concentrations and decreased serum estradiol concentrations. She was diagnosed with POI. Her case led to the identification of a homozygous Slavic founder mutation on the *NBN* gene [53]. Few other studies on *NBN* mutations causing POI exist but investigation of their specific molecular mechanisms warrants further investigation.

##### *HSF2BP* (Heat Shock Factor 2-Binding Protein)

The *HSF2BP* gene, also known as *MEILB2*, is located at 21q22.3 and its expression is restricted to germ cells and embryonic stem cells [127]. HSF2BP recruits and promotes the localisation of RAD51 and DMC1 to DSB sites and their binding to ssDNA to form nucleoprotein filaments via interaction with BRCA2 [127, 128]. In addition to interacting with BRCA2, HSF2BP also acts with transcription factors HSF2 and BNC1. Mice knocking out *Hsf2* and *Bnc1* have been reported to exhibit reduced fertility [129–131]. In the research of Marko Kallio et al., *Hsf2*-/- female mice ovulated aberrantly, with severely reduced follicle numbers at all stages, and large cystic hemorrhagic follicles were found in the ovaries of infertile mice [132].

However, most of the current reports on *HSF2BP* mutations focus on male infertility, while only a few reports of *HSF2BP* mutations resulting in female reproductive disorders. In 2020, Natalia Felipe-Medina et al. identified a homozygous missense mutation S167L on HSF2BP in three POI patients from a POI consanguineous family and the same mutation is known to cause reduced fertility and defective DNA repair in mice, evidenced by a reduction in the number of RAD51/DMC1 and the number of crossovers near DSB damage, suggesting that this homozygous missense mutation causes reduced fertility by adversely affecting HR and leading to abnormal meiosis [54]. Similarly, in 2021, Li et al. performed whole-exome sequencing of 1030 patients with sporadic POI and identified two homozygous missense mutations located on *HSF2BP*. Patients carrying both mutations had atrophied ovaries and no follicles within them and had reduced DNA damage repair, presumably due to the *HSF2BP* mutation, which causes HR defects in meiosis and results in decreased ovarian reserve and oocyte apoptosis [55]. Noticeably, *HSF2BP* mutations cause reproductive hypogonadism with relatively large variability, which often manifests as severe meiotic defects and greatly reduced spermatogenesis in males, while in females the symptoms are milder [133]and the severity varies for different female individuals. It is suggested that *HSF2BP* mutations have greater phenotypic variability between the two sexes and between individuals of the same sex, which may be related to differences in the mechanisms regulating meiotic homologous recombination and individual microenvironment between men and women.

##### *EXO1* (Exonuclease 1)

EXO1 protein is involved in DNA mismatch repair and HR [134]. In HR, EXO1 mainly exerts 5'–3' exonuclease activity to further excise the nucleotide at the 5' end of the DSB site after the MRN complex and CtIP, produce a longer 3' ssDNA tail [135, 136]. Wei et al. showed that *Exo1*-/- mice could finish the first meiotic division, but partial chromosome deletion led to meiotic failure and apoptosis. Additionally, they discovered that young adult *Exo1*-/- mice did not differ significantly from wild-type mice in terms of ovarian morphology, follicle number, or developmental stage. But as the mice grew older, the ovaries of *Exo1*-/- mice were noticeably smaller than those of wild-type littermates, and their fertility prematurely declined, exhibiting a POI phenotype similar to that of humans [137]. The *EXO1* mutation was not detected in 186 Chinese Han Chinese POI patients by Su et al. in 2016 [138], possibly due to insufficient sample size, but may also suggest that this gene has an ethnic preference for *EXO1* mutations in POI patients and *EXO1* is currently only available as a candidate gene for POI. In 2020, Luo et al. detected two novel *EXO1* heterozygous missense mutations in 50 POI patients with primary amenorrhea and demonstrated in a yeast assay that the mutations block meiotic recombination [56].

#### POI caused by genes related to homology search

##### PSMC3IP (PSMC3 Interacting Protein)

*PSMC3IP* gene, also known as *HOP2*, encodes a nuclear protein that is highly expressed in the ovary, testis, spleen and thyroid [139]. PSMC3IP tends to function together with MND1 by forming a heterodimer. In HR, this dimeric complex promotes recombinase DMC1 binding to ssDNA and enhances its recombination ability both directly and indirectly, which in turn promotes D-loop formation and strand exchange. Deletion of either protein may lead to synapsis abnormalities and programmed DSB repair errors [140–142]. As a gene definitely involved in infertility [9], five reports have identified *PSMC3IP* mutations unique to patients with POI. David Zangen et al. detected an identical *PSMC3IP* homozygous deletion mutation in all female patients of a Palestinian family. The missing glutamate residue resulted in PSMC3IP malfunction and caused HR failure, which may be associated with ovarian atrophy and reduced follicles in affected patients [139]. Abdulmoein Eid Al-Agha et al. sequenced the genes of four sisters with POI and their brother with azoospermia, from the same family and found that all five siblings carried an identical *PSMC3IP* homozygous nonsense mutation [58]. Yang et al. previously reported that a patient who had primary amenorrhea was determined to have a heterozygous loss-of-function mutation in *PSMC3IP* and had nonvisible ovaries on ultrasonographic imaging [57].Similarly, Mei et al. reported a patient with primary amenorrhea, carrying two biallelic *PSMC3IP* mutations (c.597 + 1G > T and c.268G > C), which were inherited from her biological parents. Moreover, the proband’s sister and mother also carried the c.268G > C mutation, while their reproductive function remains normal [59]. Recently, Fabio Sirchia et al. reported on patients with POI carrying a novel compound heterozygous mutation and symptoms of secondary amenorrhea [60]. Patients in the four previous reports, however, all showed primary amenorrhea, hinting that variants at different loci on PSMC3IP may induce mild or severe complications. Although there are many reports on *PSMC3IP* mutations leading to POI, there are still many questions in the study of specific molecular mechanisms, including the explanation of DSB repair errors and the quantity accumulation of protein defects, which necessitates pursuing additional experimental studies.

##### *BRCA2* (Breast Cancer Susceptibility Gene II)

BRCA2 is a tumour suppressor gene involved in HR for programmed DSBs in meiosis and is essential for maintaining genomic stability [143]. During HR, eight evolutionarily conserved motifs of the BRCA2 protein mediate the binding of ssDNA by recombinase RAD51. Only after successful disassembly of BRAC2 from RAD51, can RAD51-ssDNA nucleoprotein filaments be formed [144, 145]. Studies have shown that functionally normal BRCA2 protein is important for the maintenance of ovarian reserve levels, whereas abnormal *BRCA2* genes cause oocytes to enter the apoptotic pathway [146], leading to the acceleration of ovarian ageing [147]. Mutations in the *BRCA2* gene are known to cause a significantly increased risk of breast and ovarian cancer in women, but several studies have suggested that it is also an important gene involved in POI [9, 148]. In the study by Weinberg-Shukron et al., two sisters from a non-consanguineous family from Ethiopia had primary amenorrhea, absent normal pubertal development, abnormal hormone levels and undetectable uteri and ovaries by imaging. Sequencing revealed a compound heterozygous truncating mutation in their *BRAC2* gene, which resulted in failure of the BRAC2 protein to properly mediate the binding of RAD51 to ssDNA and impaired DSB repair leading to oocyte apoptosis. They also designed a drosophila model with knockout of the Dmbrca2 gene (the fly gene orthologous to BRCA2), which revealed that female drosophila ovaries were hypoplastic and had few mature eggs, only 4%. This suggests that BRCA2 mutation is the main cause of POI in patients [61]. Sandrine Caburet et al. Identified a homozygous hypomorphic *BRCA2* variant in POI patients without symptoms of cancer and Fanconi anaemia, while mice with a similar mutation showed germ cell depletion and loss of fertility [62]. All of the above studies have broadened the abnormal phenotype of *BRCA2* causing POI, but more studies are still needed to explore the association of the *BRCA2* gene with POI.

##### *DMC1* (Disrupted Meiotic cDNA I)

The *DMC1* gene, located at 22q13.1, was originally identified in yeast and is a homolog of the E. coli *RecA* gene [149]. Another homolog of RecA is RAD51 [150].During HR, RAD51 binds ssDNA and form nucleoprotein filaments in both mitosis and meiosis, whereas DMC1 can only participate in meiosis. The *DMC1* gene is involved in the normal synapsis of homologous chromosomes in HR and is essential for the maintenance of normal meiosis, whereas mutated *DMC1* causes prolongation of nucleoprotein filaments, the formation of abnormal recombination intermediates and abnormal meiotic products [151–153]. Mice with knockout *Dmc1* exhibit reduced ovarian size and dysplasia, with almost no follicles or oocytes in the ovaries due to apoptosis of normal eggs caused by blockage of first meiosis, causing a complete loss of reproductive capacity [149, 154]. He et al. identified a novel *DMC1* homozygous missense mutation in POI patients. This missense mutation resulted in the substitution of a conserved aspartate residue in the modified H3TH motif of DMC1 by asparagine, causing misfolding of the protein, which eventually led to the failure of HR to proceed normally, resulting in meiotic I arrest [63]. Overall, however, most POI patients with *DMC1* mutations also have a combination of other genetic mutations [103].

##### MEIOB (Meiosis-Specific with OB Domain) and SPATA22 (Spermatogenesis Associated 22)

*MEIOB* and *SPATA22* genes are meiotic-specific and are expressed only during spermatogonia and oogonia [155]. MEIOB tends to bind to SPATA22 to form a complex that is co-recruited to the DSB site, facilitating crossover formation and accurate intact synapsis [156]. *MEIOB* is a homolog of RPA1 with an OB structural domain that binds ssDNA and has 3'–5' exonuclease activity in vitro [157]. It has also been shown that the *MEIOB* mutation impairs the stability of RAD51 and DMC1 [156]. Similarly, SPATA22 binds ssDNA and promotes strand invasion. *Meiob* knockout mouse’s germ cells can initiate meiosis, but crossover and synapsis fail, resulting in unrepaired DSBs and meiotic arrest. Additionally, *Meiob*-deficient mice's ovaries were noticeably smaller and surprisingly devoid of follicles when compared to wild-type mice [65]. Moreover, due to poor development prior to meiotic pachynema, female mice with *Spata22* knockouts have severely reduced oocyte counts and quite a few of degenerated follicles in their ovaries [158].

Sandrine Caburet et al. performed whole-exome sequencing of two sisters with POI and detected a homozygous mutation in *MEIOB* that induced exon 12 skipping, leading to the production of MEIOB truncated protein [64]. The protein is unable to bind to SPATA22 and thus cannot be recruited to the DSB, resulting in meiotic failure and ultimately POI. Also worth mentioning is that this is the first report of biallelic mutation in POI. In 2021, Wu et al. identified a homozygous splicing mutation in the *MEIOB* gene in a Pakistani POI female with primary infertility, resulting in exon 9 skipping, MEIOB protein deficiency, HR failure and meiotic arrest [65]. In a recent study, Yao et al. reported the first case of *SPATA22* mutation causing POI, identifying a novel *SPATA22* homozygous mutation in a family with four siblings suffering from infertility and a compound heterozygous mutation in a patient with sporadic POI [66]. Both mutations affect the expression of the MEIOB binding domain of SPATA22, resulting in the improper binding of the two and leading to meiotic failure and infertility.

##### *SPIDR* (scaffolding protein involved in DNA repair)

The *SPIDR* gene, also known as *KIAA0146*, encodes a protein that binds to BLM or FLGNL1 to promote the aggregation of the recombinase RAD51 of HR to the damaged DSB site. This protein is also involved in stabilising recombinant intermediates and regulating the selection of HR sub-pathways [159–162]. SPIDR deletion leads to an increased probability of sister chromatid exchange, increased genomic stability and an increased incidence of hypersensitive responses to DNA damage repair [160, 163]. In 2017, Pola Smirin-Yosef et al. identified a biallelic nonsense pathogenic mutant of SPIDR in two sisters from an Israeli consanguineous family who showed delayed puberty with POI [67]. In 2022, Abdelkader Heddar et al. performed targeted next-generation sequencing of an idiopathic Indian POI patient and identified a novel *SPIDR* homozygous nonsense mutation. This mutation may lead to nonsense-mediated mRNA decay, resulting in genome instability [68]. These findings both suggest that aberrant expression of *SPIDR* is a causal factor in POI, but it has also been reported that *SPIDR* may be not an essential gene in mouse models nor in humans [67]. The accuracy of this conclusion deserves further study.

#### POI caused by genes related to intermediates processing and stabilisation

##### *MSH* (MutS Homology) *4* and *MSH5*

*MSH4* is located at 1p31.3 and *MSH5* is located at 6p21.33. MSH4 and MSH5 are homologs of bacterial MutS in eukaryotic cells [164] and often form heterodimers expressed exclusively in the gonads [165]. Unlike other MutS family proteins, MSH4 and MSH5 proteins do not participate in DNA mismatch repair, but rather bind and stabilise the Holliday intermediate produced by HR during the first meiosis and facilitate the formation of crossover [71, 166]. *Msh4* or *Msh5* knockout mice, while able to initiate meiosis normally, are unable to stabilise intermediates in the HR process and ensure proper chromosome pairing, ultimately leading to a reduced number of oocytes and signs of ovarian failure and infertility in mice [167]. Numerous familial hereditary investigations and sporadic cases have also confirmed that MSH4 and MSH5 variants can cause POI by disrupting meiosis and adversely affecting oocyte production. In a study by Carlosama et al., whole-exome sequencing of a Colombian family member with POI revealed a splice-site mutation in the *MSH4* gene that induced exon 17 skipping, leading to the elimination of the Walker B motif, which is highly conserved in the ATP-binding domain, thereby inactivating MSH4 and subsequently causing infertility [69]. Guo et al. identified a novel *MSH5* homozygous missense mutation in two sisters and later confirmed that the mutation causes infertility and reduced ovarian volume in mice with the same mutation [72]. In 2021, Arvand Akbari et al. identified a very rare *MSH4* homozygous missense mutation in a family with multiple cases of reproductive disorders that was undetected in multiple patients with sporadic POI and NOA [70]. In contrast to *MSH4*, current research on *MSH5* is focused on male reproduction [71], the specific molecular mechanisms of both leading to POI warrant further investigation.

##### *MCM* (Minichromosome Maintenance)*8* and *MCM9*

*MCM8* is located at 20p12.3 and *MCM9* is located at 6q22.31. MCM8 and MCM9 proteins belong to the minichromosome maintenance (MCM) protein family and are paralogs of MCM2 ~ 7 [168]. They polymerise to form stable heterodimers that stabilise replication forks and participate in HR as one of the helicases [169, 170]. The expression of *MCM8* is much greater in the follicular phase than in the ovulatory and luteal phases, whereas the expression levels of MCM9 had minimal variations in expression during the menstrual cycle [171]. The activity of important HR factors such as RAD51 and RPA, which stabilise ssDNA, are reduced in *Mcm8* and *Mcm9* knockout mice. This results in abnormal HR processes, almost no primary follicles and oocytes in the ovaries of the mice and an increased probability of ovarian cysts, presenting as amenorrhea and infertility [172]. Biswas et al. also demonstrated that *Mcm8-/-* mice were unable to undergo DSB damage repair and had atrophied, dysplastic ovaries, whereas *Mcm9-/-* mice exhibited mainly the absence of primary follicles [173]. In 2014, Michelle A. Wood-Trageser et al. first reported that *MCM9* mutations can cause POI. They studied two unrelated families, each with women who presented with primary amenorrhea and short stature. Whole-exome sequencing revealed that they carried different homozygous *MCM9* mutations, resulting in the production of truncated MCM9 protein and loss of protein function, respectively. The investigators hypothesised that *MCM9* mutations cause POI and reduced stature [84]. However, in the study by Fauchereau F. et al., two sisters who also had POI due to *MCM9* mutations had normal height, which expanded the *MCM9* phenotype spectrum to include patients with POI of normal stature [85]. With the rapid development of various sequencing technologies, *MCM8* and/or *MCM9* mutations have been detected in many POI patients in recent years [74, 76–79, 83]. Consequently, these two genes can be identified as common causative genes of POI, and their mutations may result in the failure of MCM8 and MCM9 proteins to be accurately recruited to the DSBs locus, decreasing the efficiency of HR and greatly reducing the number of oocytes that can complete meiosis. The exact molecular structures of MCM8 and MCM9 are yet unknown, but their complicated architectures are expected to explain how they function as helicases in HR and how POI develops molecularly.

##### HFM1 (Helicase for Meiosis 1)

The *HFM1* gene, also called *MER3*, is mainly expressed in germ cells and its encoded protein is an ATP-dependent helicase [174] involved in homologous chromosome crossing over and synapsis in meiosis [175, 176]. This gene has been identified as one of the causative genes for POI. Mice with *Hfm1* mutations exhibit reduced follicular reserve and reduced egg quality leading to infertility or subfertility [174, 177]. While mice lacking the *Hfm1* gene had significantly smaller ovaries, with fewer follicles and corpus luteum. Studies have demonstrated that *Hfm1* mutations affect the mid- to late-stages of HR, impairing folliculogenesis and causing infertility by reducing crossovers and failing to synapsis [176]. In 2014, Wang et al. identified a compound heterozygous mutation in the *HFM1* gene in two Chinese sisters with POI and subsequently screened for *HFM1* mutations in 69 Chinese women who had sporadic POI, leading to the identification of another patient with a compound heterozygous mutation [90]. Both this experimental study and the 2016 study by Pu et al. found significantly higher *HFM1* variability in the POI group than in the control group [91]. In 2019, Zhe et al. identified a novel heterozygous missense mutation in the *HFM1* gene, which causes mRNA splicing defects, in two POI patients from a Chinese family. Both patients developed ovarian dysfunction before age 40 years, while a mouse model with the same mutation exhibited significantly lower follicle numbers at all levels as well as ovarian atrophy [92]. In recent years, new types of *HFM1* mutations have been identified in POI patients [89, 103], including some patients with multiple mutations including *HFM1*. This provides an opportunity to further explore the role of HFM1 mutations in the development of POI, including the impact mutations have on HR.

##### *ERCC6* (Excision Repair Cross-Complementation Group 6) and *PGBD3* (PiggyBac Transposable Element Derived 3)

The *ERCC6* gene, also called *CSB* gene, is located at 10q11.23 and belongs to the SWI2/SNF2 superfamily [178, 179]. The ERCC6 protein is required for transcription-coupled nucleotide excision repair and is also associated with chromatin remodelling, intermittent cross-link repair, telomere maintenance and transcription-related DNA recombination [180]. ERCC6 has been found to be involved in DSB damage repair, mainly through its winged helix domain interacting with RIF1, promoting the recruitment of RPA, RAD51 and BRCA51 to DSB damage sites and participating in DNA end resection. This allows it to control the choice of DSB repair pathways, boosting HR while suppressing NHEJ [178, 179, 181, 182]. Defects in the *ERCC6* gene are known to be associated with a variety of diseases, such as Cockayne Syndrome B, retinal dystrophies, arrhythmia and immunodeficiencies [183–185]. Additionally, studies have documented cases of *CSB-PGBD3* fusion gene mutations leading to POI prior to reports of simple *ERCC6* mutations that cause POI. In 2015, Qin et al. first identified three novel CSB-PGBD3 fusion protein mutations (c.2237G > A: p.G746D, c.643G > T: p.E215X, c.3166G > A: p.V1056I). Among them, a p.G746D mutation was found to be inherited in an autosomal dominant manner in non-consanguineous families, while a new missense mutation p.V1056I and a nonsense mutation p.E215X were found in 432 sporadic POI cases. All three mutations interfere with DNA damage repair and affect normal ovarian development resulting in POI [93]. In 2021, Shen et al. identified a novel heterozygous mutation in *ERCC6* in a patient with idiopathic POI, which was derived from the patient's phenotypically normal father, but was not found in the mother and sister, who were also phenotypically normal [74], suggesting that the genotype is consistent with a paternal mode of inheritance. The specific mechanisms of *ERCC6* gene and *CSB-PGBD3* fusion gene mutations in the pathogenesis of POI should be further investigated in the future.

#### POI caused by genes related to resolution of recombination intermediates

Proper segregation of late HR chromosomes requires a series of protein processing recombinant intermediates, which include MLH1-MLH3, SLX1-SLX4, MUS81-EME1 dimers and BTR complexes, GEN1, etc. [186–188]. Although mutations in these proteins have been reported to cause infertility in mice [189, 190], the mice did not exhibit POI-like symptoms. Only in 2018, Sunita Katari et al. reported the presence of a partial duplication of the MLH1 gene in a POI patient who developed secondary amenorrhea at age 31 years, resulting in an encoded protein with a missing ATPase domain. However, this patient also had a combination of other familial genetic disorders with multiple chromosome breaks [191], indicating that her abnormal *MLH1* gene duplication may not be a contributing cause of her secondary amenorrhea and *MLH1* gene is also a candidate for POI.

### POI caused by genes related to NHEJ

NHEJ is not only the main method of DSB repair, but is also involved in V(D)J rearrangement and class switch recombination of lymphocytes, which are essential for T and B cell maturation. Defects in V(D)J rearrangement mechanisms lead to a decrease in the number of T and B cells in the body and exhibit severe immunodeficiency [192]. Mutations in the NHEJ pathway often lead to skeletal malformations, immune deficiencies and a short life expectancy in most patients [193]. This has led to a limited number of studies on *NHEJ*-related genetic mutations and their impact on gonadal development and reproduction. A very small number of patients have survived to adulthood and all have exhibited abnormal reproductive function.

In a recent report, Li et al. performed whole-exome sequencing of a family with three generations of POI patients and identified a rare heterozygous natural loss-of-function mutation located in *NHEJ1* [113]. NHEJ1 binds XRCC4-LIG4 complex, stabilises and enhances the ligating activity of the complex and may also mediate the recruitment of other NHEJ components, such as Ku and DNA-PKcs [194, 195]. Mice with the same mutation have remarkably fewer follicles and a 50% reduction in ovarian size compared to wild-type mice Breeding tests illustrated that mice with the *Nhej1* mutation take longer to complete their first birth, demonstrating that the mutation causes reduced fertility. In this study by Li et al., another *NHEJ1*-related missense mutation identified from the analysis of 100 patients with sporadic POI was also shown to cause DNA repair defects and ovarian decline with functional abnormalities such as immune deficiency, increased susceptibility to DNA damage and DSB repair defects [113]. In addition to *NHEJ1*, mutations in *DNA-PKcs*, *LIG4*, *XRCC4* and other important NHEJ pathway genes have also been reported to cause primary gonadal failure [196–198].

### POI caused by genes related to cohesin complex

#### *STAG3* (Stromal Antigen 3)

STAG3 maintains the stability of the cohesin complex, ensures proper synapsis and segregation of homologous chromosomes and promotes the production of haploid gametes [199, 200]. It has been shown that both male and female mice knocked out of *Stag3*, are sterile, which may be related to the failure of homologous chromosome synapsis and meiotic arrest caused by the mutated protein [201]. Mice with a homozygous *Stag3* mutation are characterised by having almost no follicles, reduced ovarian volumes and infertility [202]. *STAG3* mutations are not uncommon in genetic sequencing studies of POI patients in recent years. In 2014, Sandrine Caburet et al. detected a *STAG3* homozygous splicing mutation in all POI patients in a highly consanguineous family [94]. Polona Le Quesne Stabej et al. reported two sisters from Lebanon with primary amenorrhea and absence of pubertal development carried a homozygous *STAG3* duplication mutation, resulting in a loss of protein function [95]. Xiao et al. reported two sisters with symptoms of small uteri and ovaries and who are infertile, both carry two novel *STAG3* homozygous frameshift mutations [99]. In 2020, Jaillard et al. detected a novel *STAG3* homozygous missense mutation in a patient with POI. Further studies revealed that her brother also carried the mutation and was diagnosed with NOA. Moreover, the proband presented with multiple developmental abnormalities in the skeleton, secondary sexual characteristics, ovaries, and mammary glands, as well as hormone level disturbances [100]. Recently, Arvand Akbari et al. reported three POI and NOA patients from Iranian families of consanguineous marriages. Sequencing revealed that all three of the siblings carry two *STAG3* homozygous mutations, resulting in in-frame deletion and amino acid substitution, respectively [105]. Susana et al. reported a 16-year-old patient with symptoms of primary amenorrhea, pubertal dysplasia and genital hypoplasia. Whole-exome sequencing results showed that she carried a novel *STAG3* homozygous deletion mutation [106].

### REC8 (REC8 Meiotic Recombination Protein) and RAD21L (RAD21 Cohesin Complex Component Like 1)

Both REC8 and RAD21L belong to the a-kleisin subunit of cohesin complex and both can interact with other subunits such as SMC and STAG3 to promote sister chromatid separation. Male germ cells with a *RAD21L* mutation leads to synapsis failure and meiotic arrest, causing male infertility, while females with a *RAD21L* mutation remain fertile. As women age, however, the function of *RAD21L* in maintaining female fertility becomes increasingly important [203]. Therefore, although the *RAD21L* mutation has not been reported in POI patients, it remains a potential causative gene for POI after age 30.

Huiling Xu et al. demonstrated that synapsis abnormalities in mice with deletion of *Rec8* occurred between sister chromatids, which may be associated with high early mortality, premature gonadal failure and infertility in mice [204]. In 2016, Justine Bouilly et al. randomised 100 patients with POI and identified two *REC8* missense mutations in three patients by next-generation sequencing. The first two patients had symptoms of primary amenorrhea, underdeveloped secondary sexual characteristics and a combination of other POI susceptibility gene mutations. The other patient had secondary amenorrhea and only one *REC8* mutation [108]. However, a study by Tuckerd et al. in 2021 showed that a biallelic loss-of-function *REC8* mutation resulted in a secondary infertility phenotype, whereas her mother, who carried the *REC8* single locus mutation, had no reproductive deficiency condition. This suggests that heterozygous *REC8* mutations do not cause POI and that POI triggered by *REC8* biallelic mutations may be associated with autosomal recessive inheritance [107].

### *SMC1B* (Structural Maintenance of Chromosomes 1B)

SMC1B promotes the formation of axial elements of synaptonemal complexes, which affects follicle formation once mutated [205]. Male and female mice knocked out of *Smc1b* exhibit different degrees of meiotic defects, such as incomplete homologous chromosome synapsis, premature deletion of cohesin complex proteins between sister chromatids and reduced number of crossover-associated recombination foci and both sexes are sterile [206]. In 2009, Shuji Takabayashi et al. identified a spontaneous homozygous *Smc1b* splicing mutation in mice that caused a significant reduction in the number of oocytes in female mice and caused infertility [207]. Justine Bouilly et al. identified two *SMC1B* missense mutations in two patients who presented with primary and secondary amenorrhea, respectively, however, they were also found to have additional gene mutations [108]. Moreover, there is no evidence that *SMC1B* mutations alone can cause POI, so SMC1B is only a candidate gene, requiring deeper research in this area later.

### POI caused by genes related to synaptonemal complex

#### *C14orf39* (Chromosome 14 Open Reading Frame 39)

C14orf39 is a protein contributing to the central element of the synaptonemal complex, also known as Six6os1 in mice. *Six60s1* promotes SC formation and chromosomal synapsis by interacting with the synaptonemal complex central element 1 (SYCE1). Mice deficient in *Six60s1* have defective chromosome synapsis at prophase I of meiosis, triggering an arrest at the pachytene stage and exhibiting massive oocyte apoptosis [208]. In 2021, Fan et al. identified a homozygous *C14orf39* frameshift mutation in three siblings with POI, of a consanguineous Pakistani family. The frameshift mutation may result in the production of a truncated protein that completely blocks homologous chromosome synapsis and causes oocyte apoptosis [109]. A recent report identified a novel *C14orf39* biallelic nonsense mutation in two patients with sporadic POI, both of whom presented with secondary amenorrhea, no history of pregnancy, small ovarian morphology with minimal to no follicles on ultrasonographic examination [110]. Given that Fan et al. also found that two unrelated sterile males carrying homozygous nonsense mutations or splicing mutations in *C14orf39* were the primary cause of their infertility [109], we can tentatively speculate that *C14orf39* often triggers POI or other infertility problems in the form of homozygous mutations.

### *SYCE1* (Synaptonemal Complex Central Element Protein 1)

SYCE1 is a protein that contributes to the CE of the synaptonemal complex. Mice with *Syce1* knocked out show oocyte apoptosis and POI-like symptoms due to blocked DSB repair in meiosis [209]. The study by Sanchez-Saez et al. further demonstrates that although *Syce1-/-* mice can produce programmed DSBs, both failure of SC assembly and defective crossover formation lead to HR failure, resulting in follicular apoptosis in female mice [210]. In 2011, McGuire and his colleagues found a heterozygous microdeletion of *SYCE1* in one of 89 POI patients by SNP microarray technology, and the patient was amenorrheic at age 21 [211]. In 2014, Liat de Vries et al. reported a case of POI due to a homozygous nonsense mutation in *SYCE1*. The two sisters affected were from a consanguineous Israeli Arab family and both presented with primary amenorrhea symptoms, extremely low serum oestrogen levels and almost no detectable follicles [111]. In 2020, Diego Hernández-López et al. used CRISPR/Cas9 technology to produce a humanised mouse model that allowed the mouse to carry the *Syce1* homozygous mutation identified by Liat de Vries et al., which recreated the infertility phenotype. Compared to wild-type or heterozygous mice, mice carrying the homozygous mutation are infertile with poorly developed ovaries and no follicles. In addition, the team detected almost no production of SYCE1 protein in mice carrying the homozygous *Syce1* mutation, suggesting that the mutation resulting in greatly reduced transcriptional activity of *Syce1* is one of the key mechanisms leading to infertility [212].

Additionally, two sisters from a consanguineous family were detected with a *SYCE1* homozygous deletion mutation and exhibited secondary amenorrhea and elevated serum gonadotropin levels. The mutation alters the primary structure of the SYCE1 protein, causing extreme instability in the SC structure formed during prophase meiosis, which mismatches homologous chromosomes and prevents the formation of crossovers, ultimately leading to meiotic arrest [112]. In addition to familial POI, Hou et al. detected a novel *SYCE1* biallelic mutation in 1030 patients with sporadic POI [110]. It has also been shown that *Syce1-/-* female mice display a similar meiotic prophase phenotype to that of males, suggesting that *SYCE1* plays the same role in male and female meiosis [209]. From previous studies, it is not difficult to find a wide phenotypic variability in *SYCE1* mutations. Homozygous *SYCE1* mutations with obvious family inheritance may lead to primary amenorrhea or secondary amenorrhea. In contrast, heterozygous mutations with only sequence microdeletions result in a secondary infertility phenotype, while patients suffer from menorrhagia prematurely and are equally afflicted with this mutation. We speculate that it may be due to the fact that mutations at different loci of *SYCE1* produce proteins with widely differing structure and function, which result in different stability of SC, further creating differences in the rate of successful homologous chromosome pairing and the number of gametes formed, thus leading patients to exhibit different infertility phenotypes.

## Summary and future perspectives

This review summarizes the DSB formation and damage repair genes associated with the pathogenesis of POI that have been identified in recent years, including genes that have widely been acknowledged to cause POI (HSF2BP、PSMC3IP、BRCA2、SPIDR、MSH4、MSH5、MCM8、MCM9、HFM1、ERCC6、STAG3、C14orf39、SYCE1), as well as candidate genes that are less significant in the development of POI and have not been confirmed to cause POI (PRDM9、ANKRD31、NBN、EXO1、DMC1、MEIOB、SPATA22、REC8、RAD21L、SMC1B、MLH1、NHEJ1). POI is a highly heterogeneous disease associated with impaired fertility in women of reproductive age and severely impacting the physical and mental health of affected women. POI may also lead to familial and social discord due to unrealised expectations both regarding impaired development of secondary sex traits and impaired fertility. Multiple genetic and immune factors are currently known to cause POI, yet these known etiologies still do not explain the specific causative mechanisms in the majority of clinical patients. While researchers have identified a large number of key proteins in the DSB repair pathway and have confirmed through mouse models that abnormal expression of these proteins leads to follicular reduction and oocyte apoptosis due to DSB repair failure resulting in POI; the specific molecular mechanisms and related signalling pathways are still largely unknown.

Female reproductive health and the development of POI present a vast array of future research endeavours which stands to benefit all women as well as the worldwide medical community. As an increasing number of couples delay marriage too much later in their lives than their predecessors had a decade or more ago, leading to an increasing number of women engaging in childbirth after age 30. The incidence of POI is about 1% between the ages of 30 and 40, making it especially important to prevent and detect POI earlier in the woman’s life. As more mutations are discovered with the advanced techniques currently available, POI patients who test positive for a mutation could lead to screening their relatives for the same mutations with genetic sequencing. If the test results are positive, a more thorough evaluation of reproductive function, including follicle and granulosa cell counts, hormone levels like FSH and AMH, and the existence of other illnesses that affect reproductive function, ought to be carried out. Accordingly, specialized centers that deal with infertility should develop individualized strategies to prevent or treat infertility for such people, which might involve techniques based on mesenchymal stem cells, ovarian tissue, oocyte or embryo cryopreservation, primordial follicle in vitro activation, etc. However, there is currently no standardized, unambiguous preventive and treatment regimen for POI caused by genetic disorders, making it challenging to suggest the best course of action. There is no doubt, however, that proactive avoidance as well as early discovery, diagnosis, and therapy can be extremely helpful in maintaining fertility in individuals with POI brought on by genetic causes.

## Abbreviations

POI: Premature ovarian insufficiency
DSB: DNA double-strand break
HR: Homologous recombination
NHEJ: Non-homologous end joining
*PRDM9*: PR-Domain Containing Protein 9
*ANKRD31*: Ankyrin Repeat Domain 31
*NBN*: Nibrin
*HSF2BP*: Heat Shock Factor 2-Binding Protein
*EXO1*: Exonuclease 1
PSMC3IP: PSMC3 Interacting Protein
*BRCA2*: Breast Cancer Susceptibility Gene II
*DMC1*: Disrupted Meiotic cDNA I
MEIOB: Meiosis-Specific with OB Domain
SPATA22: Spermatogenesis Associated 22
*SPIDR*: Scaffolding protein involved in DNA repair
*MSH*: MutS Homology 4
*MSH5*: MutS Homology 5
*MCM*: Minichromosome Maintenance *8*
*MCM9*: Minichromosome Maintenance 9
HFM1: Helicase for Meiosis 1
*ERCC6*: Excision Repair Cross-Complementation Group 6
*STAG3*: Stromal Antigen 3
*REC8*: REC8 Meiotic Recombination Protein
*RAD21L*: RAD21 Cohesin Complex Component Like 1
*SMC1B*: Structural Maintenance of Chromosomes 1B
SC: Synaptonemal complex
*C14orf39*: Chromosome 14 Open Reading Frame 39
LE: Lateral elements
CE: Central element
TF: Transverse filaments
*SYCE1*: Synaptonemal Complex Central Element Protein 1
